# Lipid Composition in the Breast of Premenopausal *BRCA1/2* Genetic Mutation Carriers Using MRI

**DOI:** 10.3390/ijms27198472

**Published:** 2026-09-23

**Authors:** Sai Man Cheung, Kwok-Shing Chan, Senthil Ragupathy, Zosia Miedzybrodzka, Jiabao He

**Affiliations:** 1 Medical Physics & Engineering, The Leeds Teaching Hospitals NHS Trust, Leeds LS9 7TF, UK; 2 Athinoula A. Martinos Center for Biomedical Imaging, Massachusetts General Hospital, Charlestown, MA 02129, USA; 3 Department of Radiology, Harvard Medical School, Boston, MA 02115, USA; 4 Department of Radiology, Aberdeen Royal Infirmary, Aberdeen AB25 2ZN, UK; 5 Institute of Medical Sciences, School of Medicine, Medical Sciences and Nutrition, University of Aberdeen, Aberdeen AB25 2ZD, UK; 6 Newcastle Magnetic Resonance Centre, Translational and Clinical Research Institute, Faculty of Medical Sciences, Newcastle University, Newcastle upon Tyne NE4 5PL, UK

**Keywords:** *BRCA1/2*, chemical shift-encoded imaging, monounsaturated fatty acids, polyunsaturated fatty acids, saturated fatty acids

## Abstract

Deregulation of lipid composition in the breast has been suggested as an indicator of the risk of developing breast cancer, and chemical shift-encoded imaging (CSEI) in MRI allows rapid lipid composition mapping of the whole breast. We set out to examine if breast lipid composition in *BRCA1* and *BRCA2* gene carriers differed from women with breast cancer and controls. Eighty-two premenopausal female participants, 30 *BRCA1/2* carriers, 37 women with breast cancer, and 15 controls, participated in this study. Monounsaturated, polyunsaturated, and saturated fatty acids (MUFAs, PUFAs, SFAs) in the breast of *BRCA1/2* carriers, women with breast cancer, and controls, were acquired using CSEI. The coefficient of variation and intraclass correlation coefficient were calculated for intra-session repeatability and 6-/12-month test–retest reliability, respectively. There was a significantly higher proportion of MUFAs (*p* = 0.01) in the breast of *BRCA1/2* carriers (median: 0.42, IQR: (0.41–0.43)) compared with controls (0.41, (0.41–0.42)). There was a significantly lower proportion of SFAs (*p* = 0.01) in *BRCA1/2* carriers (0.44, (0.42–0.46)) compared with controls (0.46, (0.45–0.48)). However, there was no significant difference in the proportions of MUFAs, PUFAs, and SFAs between *BRCA1/2* carriers and women with breast cancer. The coefficients of variation were below 10.0% for all lipid constituents. The intraclass correlation coefficients for MUFAs, PUFAs, and SFAs were 0.80, 0.84, and 0.80 at 6 month, and 0.75, 0.77, and 0.78 at 12 month, respectively. The deregulation in lipid composition of the breast has a potential for risk stratification and early detection of breast cancer in a high-risk population.

## 1. Introduction

Breast cancer is a major and expanding health challenge, and the introduction of screening service and new drugs have significantly improved the survival rate [1]. In the UK, patients with stage 1 breast cancer have almost 100% 5-year survival, compared with up to 90% and 70% for stage 2 and 3 breast cancer, respectively [2], highlighting the critical need of improving early detection. Germline pathogenic variants in the genes *BRCA1* and *BRCA2* are associated with very high lifetime breast cancer risk [3,4]. The prevalence of germline *BRCA1/2* mutated genes in the general population has generally been quoted as approximately 1 in 800 [5], but a population-based mammographic screening program in the UK has reported rates as high as 1 in 400 [6], with higher rates in the populations of Ashkenazi Jews or Eastern Europeans [5]. Since 2013, the National Institute for Health and Care Excellence (NICE) has recommended that premenopausal *BRCA1/2* genetic mutation carriers are offered the options of risk-reducing mastectomy or annual surveillance using dynamic contrast-enhanced (DCE)-MRI [7], the current most sensitive imaging modality with a sensitivity of over 94% for a diagnosis of breast cancer in dense breast [8]. However, DCE-MRI only highlights tumour angiogenesis in malignancies that are well under development, and the impact of long-term residual contrast agent retention in the brain [9] and toxicity in patients with kidney disease [10] remain a concern.

The breast is largely composed of fat, and alternations of lipid metabolism in breast tissues of *BRCA* gene carriers has been documented [11,12,13]. A nine-node query (*BRCA1*, *BRCA2*, *MYC*, *PTEN*, *TP53*, *ACACA*, *FABP4*, *FASN*, *SCD*) on STRING [14] indicated loss of *PTEN* expression with *p53* deficiency and *BRCA1/2* mutations lead to co-amplification of *MYC* correlated with tumour heterogeneity and cell growth. Pathogenic variants in the *BRCA1* and *BRCA2* genes cooperate with deletion of *PTEN* to alter phospholipid metabolism critical for cellular membrane synthesis [13]. *BRCA1* mutation disrupts the *BRCA1*-acetyl-CoA carboxylase alpha (*ACACA*) complex, inducing de novo lipogenesis and a critical change in the lipid metabolism of monounsaturated fatty acids (MUFAs) and saturated fatty acids (SFAs), leading to improved survival in breast cancer cells [11,12] and breast cancer xenografts [15,16]. *BRCA2* mutation inhibits the binding site of oestrogen receptors, impairing signal transmission of oestrogen [17,18] to fatty acid synthase (*FASN*) and stearoyl-CoA desaturase-1 (*SCD*) critical for lipid metabolism, leading to the deregulation of the MUFA-to-SFA ratio [19]. *BRCA2* mutant tumours modulate the tumour microenvironment to evade immune detection and clearance and drive polyunsaturated fatty acids (PUFAs) to activate fatty acid binding protein 4 (*FABP4*) to upregulate tumour-associated macrophages and endothelial cells [20,21], exacerbating tumour-promoting inflammation and accelerating breast cancer progression and metastasis [22,23]. The switch in lipid metabolism suggests that lipid composition might be altered with potential for imaging-derived lipid phenotypes in association with *BRCA1/2* carrier status. Deregulation of lipid composition, including MUFAs, PUFAs, and SFAs, has been shown in the breast of *BRCA1/2* carriers using single voxel spectral-edited magnetic resonance spectroscopy [24]; however, this method is susceptible to partial volume effect and voxel location selection in heterogeneous breast, with low reproducibility. We have previously shown that novel chemical shift-encoded imaging (CSEI) allows rapid lipid composition mapping of the whole breast [25,26], and the improved spatial coverage reduces partial sampling error, leading to higher accuracy with potential for early detection and response in oestrogen receptor-positive breast cancer [25] and patients undergoing chemotherapy [26].

We therefore hypothesise that lipid composition in the whole breast of young *BRCA1/2* carriers will support early detection for the high-risk population with an elevated risk of developing breast cancer, and lipid composition quantified using CSEI is highly reproducible to facilitate active surveillance.

## 2. Results

A prospective longitudinal study was conducted to compare lipid composition in the breast adipose tissue of women with *BRCA1/2* genetic mutation, women with breast cancer, and healthy controls (Figure 1).

There was no significant difference in age, breast density, body mass index, exercise MET minutes between *BRCA1/2* genetic mutation carriers, women with breast cancer, and controls (Table 1), and the histopathological findings of women with breast cancer are shown in Table 2.

There was a significantly higher proportion of MUFAs in the whole breast of *BRCA1/2* carriers (median: 0.42, IQR: 0.41–0.43; *p* = 0.01) compared with controls (0.41, 0.41–0.42) (Figure 2, Table 3). There was a significantly lower proportion of SFAs in the whole breast of *BRCA1/2* carriers (0.44, 0.42–0.46; *p* = 0.01) compared with controls (0.46, 0.45–0.48) (Figure 2, Table 3). There was no significant difference in the proportion of PUFAs (*p* = 0.03) between *BRCA1/2* carriers and controls (Figure 2, Table 3). There was no significant difference in the proportions of MUFAs (*p* = 0.10), PUFAs (*p* = 0.06), and SFAs (*p* = 0.05) between the whole breast of *BRCA1/2* carriers and the whole breast of women with breast cancer (Figure 2, Table 3). There was no significant difference in the proportions of MUFAs (*p* = 0.02), PUFAs (*p* = 0.31), and SFAs (*p* = 0.36) between the whole breast of *BRCA1/2* carriers and the peri-tumoural region in women with breast cancer (Figure 2, Table 3). There was a significantly lower proportion of MUFAs in the peri-tumoural region (0.41, 0.37–0.42; *p* = 0.008) compared with the whole breast of women with breast cancer (0.43, 0.42–0.44) (Figure 2, Table 3). There was a significantly lower proportion of PUFAs in the peri-tumoural region (0.13, 0.10–0.15; *p* = 0.008) compared with the whole breast of women with breast cancer (0.15, 0.13–0.17) (Figure 2, Table 3). There was a significantly higher proportion of SFAs in the peri-tumoural region (0.46, 0.41–0.51; *p* = 0.006) compared with the whole breast of women with breast cancer (0.42, 0.39–0.45) (Figure 2, Table 3).

For intra-session repeatability, the coefficients of variation were below 10.0% for all lipid constituents in the participants (Supplementary Figure S1, Supplementary Table S1). For test–retest reliability, the intraclass correlation coefficients for MUFAs, PUFAs, and SFAs were 0.80 (95% CI: 0.75–0.88), 0.84 (0.78–0.91), and 0.80 (0.75–0.88) at 6-month, and 0.75 (0.62–0.85), 0.77 (0.60–0.94), and 0.78 (0.52–0.93) at 12-month, respectively (Supplementary Figure S2).

## 3. Discussion

In this study, we measured lipid composition in premenopausal *BRCA1/2* genetic mutation carriers, women with breast cancer, and healthy controls using CSEI, and found a significant higher proportion of MUFAs and a lower proportion of SFAs in the breast of *BRCA1/2* carriers in comparison to controls, but no significant difference between *BRCA1/2* carriers and the peri-tumoural region or the whole breast of women with breast cancer. There was a significant difference in the proportions of MUFAs, PUFAs, and SFAs between the peri-tumoural region and the whole breast in women with breast cancer. CSEI had high intra-session repeatability and good 6-month and moderate 12-month test–retest reliability in the observed lipid composition of the breast.

The higher proportion of MUFAs and lower proportion of SFAs in the breast of *BRCA1/2* carriers in comparison to controls were in agreement with a previous study conducted using specialist magnetic resonance spectroscopy [24]. *BRCA1* transfection has been shown to impact the lipid metabolism of MUFAs and SFAs in breast cancer animal models [15,16]. *BRCA2* mutation has been shown to impair the efficiency of *FASN* and *SCD* [17,18] in lipid metabolism, altering the ratio of MUFAs to SFAs in human mammary cell lines [19]. The difference in the lipid composition of the breast in *BRCA1/2* carriers from controls, but not from women with breast cancer, indicated the potential of lipid composition as imaging-derived lipid phenotypes in association with *BRCA1/2* carriers. An increase in MUFAs and PUFAs in lipid composition in association with an immune response in women with breast cancer after one cycle of neoadjuvant chemotherapy has been shown by us [26], and a deregulation in MUFAs and PUFAs in lipid metabolism subsequent to an upregulation of endothelial cells and inflammation [20,21] might present a sensitive surrogate marker for early detection of mutant *BRCA1/2*-induced immune response. Our study is the first to show a significant change in lipid composition from the healthy to high-risk group, underscoring the importance of screening for lipid composition in high-risk populations that may lead to early detection of sporadic breast cancer, facilitating risk stratification and identification of prognostic markers [27,28].

The lower proportions of MUFAs and PUFAs and higher proportion of SFAs in lipid composition in the peri-tumoural region in comparison to the whole breast in women with breast cancer were also observed in our previous work in a UK population of postmenopausal women with oestrogen receptor-positive breast cancer [25], indicating lipid composition in association with tumour phenotypes despite menopausal status. Lipid trafficking of MUFAs and PUFAs in lipid metabolism from the peri-tumoural region to the tumour supports accelerated membrane synthesis [29,30] and pro-inflammatory activities in the tumour microenvironment [31,32], respectively. The removal of SFAs from the tumour core alleviates lipotoxicity to improve tumour survival advantage and promotes membrane lipid saturation to enhance resistance to chemotherapy [33,34]. The significant difference in the proportions of MUFAs, PUFAs, and SFAs in lipid composition between the peri-tumoural region and the whole breast in women with breast cancer indicated the potential of lipid composition for the characterisation of early breast cancer, although our results could not directly demonstrate the activation of specific metabolic pathways.

The coefficients of variation of lipid constituents in all the regions of interest and participants were below 10%, indicating high intra-session repeatability and robustness of the method. The overall test–retest reliability of the lipid composition map was moderate to good [35], indicating that the deregulation in lipid composition of the breast was a fundamental characteristic trait of *BRCA1/2* genetic mutation. The high consistency of the outcome measures is crucial for effective surveillance of *BRCA1/2* genetic mutation carriers, indicating that an isolated alteration in lipid composition is independent of transient factors such as diet.

A key strength of this study was the identification of surrogate markers sensitive to early disease change with immediate clinical impact [28]. The prospective study included a reasonably large cohort size of a high-risk population with age-matched healthy controls and women with breast cancer. Our study is the first investigation of lipid composition in the whole breast adipose tissue of *BRCA1/2* carriers, providing early evidence in the effective early detection for potential reduction in the incidence of breast cancer of poor prognosis [28]. Strict inclusion criteria were used to recruit a homogeneous cohort in each arm to minimise the impact of potential confounding factors [27,28], including hormones, exercise, and body mass. All patients with breast cancer in our cohort were not from the high-risk clinic and had no first-relative family history of breast cancer, minimising the possibility of inclusion of *BRCA1/2* gene carriers in the patient group. The results from group comparisons, together with repeatability and test–retest reliability, will inform the confidence range and variability in future implementations of potential individualised early detection strategies in parallel to DCE-MRI in the clinical settings.

One of the limitations in the current study was the limited number of participants. The current study was a relatively large prospective study; however, future large cohort and multi-centre studies are required to demonstrate the effectiveness of lipid composition in the breast as a biomarker for early detection. This investigation supports the determination of upper and lower limits of normal for lipid composition in a mixed group of a high-risk population, and future characterisation of lipid composition with respect to *BRCA1* and *BRCA2* would support the investigation of the individual disease trajectories between the two genotypes [36]. This study presents relative lipid fractions without direct measurements of absolute lipid concentrations or metabolic activity; however, alteration in one lipid fraction might influence the relative proportion of others, and future validation in tissues would be required to support the observed alterations in lipid composition in relation to lipid metabolism. This study included few participants with mammographic dense breast, and the clinical utility of lipid composition in participants with high mammographic breast density should be examined in a future study [37]. Lipid composition has been identified as a characteristic marker in *BRCA1/2* carriers and women with invasive ductal carcinoma, and further work is required to study other high-risk populations and cancer phenotypes.

## 4. Materials and Methods

To test the hypothesis, a prospective longitudinal study was conducted between March 2022 and May 2024, to compare the lipid composition in the breast adipose tissue of women with *BRCA1/2* genetic mutation, women with breast cancer, and healthy controls. This study was approved by the East of England—Essex Research Ethics Committee (identifier: 22/EE/0020), and signed written informed consent was obtained from all participants.

### 4.1. Clinical Procedure

Eighty-two premenopausal female participants, comprising 30 *BRCA1/2* genetic mutation carriers (age: [mean] 45.1 ± [SD] 8.3 years), 37 women with invasive ductal carcinoma of the breast (45.6 ± 7.0 years), and 15 age-matched healthy controls (42.7 ± 7.2 years), took part in this study. Premenopausal women aged 30–55 years with germline pathogenic variants in *BRCA1* or *BRCA2* being invited for MRI breast screening by National Health Service Grampian and women receiving a new diagnosis of breast cancer in the breast unit at Aberdeen Royal Infirmary were invited to participate. Symptomatic patients were not from the high-risk clinic and were screened for first-relative family history of breast cancer. Healthy controls were identified through university staff email and word of mouth at the University of Aberdeen. Women undergoing primary breast cancer surgery with breast conservation (tumour size larger than 10 mm in diameter on mammography), with no previous malignancies, hormonal therapy, chemotherapy, or radiotherapy prior to surgery were eligible. Participants with diabetes or on statins or cholesterol control drugs were not eligible. Controls were not at population risk of breast cancer or at high risk, including benign lesions under surveillance or body mass index higher than 30 (Figure 1). Breast density was not controlled during recruitment, and subsequently 1 woman with breast cancer, 0 *BRCA1/2* genetic mutation carriers, and 2 controls with dense breast were included. A total of 170 scans were conducted, with 82 scans for cross-sectional study, 58 scans for intra-session repeatability, and 30 scans for *BRCA1/2* carriers for 6- and 12-month test–retest reliability. All participants filled in the International Physical Activity Questionnaire (IPAQ) to estimate the exercise metabolic equivalent task (MET) minutes for the week preceding the scan [38]. All MRI images were reported by a radiologist with over 15 years of experience in breast MRI. Standard clinical histopathological examination was performed in women with breast cancer to determine histological tumour size and grade [39].

### 4.2. Magnetic Resonance Imaging (MRI)

All images were acquired on a 3 T whole-body clinical MRI scanner (Ingenia dStream, Philips Healthcare, Best, Netherlands), using a 16-channel breast coil for signal detection and a body coil for uniform transmission. Lipid composition images were acquired from all participants using a multi-slice CSEI sequence [25,26] with 16 echoes, an initial echo time of 1.14 ms, echo spacing of 1.29 ms, repetition time of 60 ms, matrix size of 176 × 176, pixel size of 2.0 × 2.0 mm^2^, slice thickness of 3 mm, and acquisition time of 5.0 min. Diffusion-weighted and dynamic contrast-enhanced images were acquired from *BRCA1/2* genetic mutation carriers and in women with breast cancer as part of the standard screening protocol, and were used in this study for tumour localisation and delineation. Diffusion-weighted images were acquired using a pulsed gradient spin echo sequence, with two diffusion weightings of 0 and 800 s/mm^2^, echo time of 50 ms, repetition time of 4000 ms, matrix size of 176 × 176, pixel size of 2.0 × 2.0 mm^2^, and slice thickness of 6 mm [25,26]. Dynamic contrast-enhanced images were acquired using a fast field echo sequence, with an echo time of 2.3 ms, repetition time of 3.9 ms, matrix size of 528 × 528, pixel size of 0.67 × 0.67 mm^2^, and slice thickness of 1.5 mm.

Image analysis was conducted in MATLAB (R2020a, MathWorks Inc., Natick, MA, USA) and ImageJ (v1.52p, National Institute of Health, Bethesda, MD, USA) [40]. The maps showing the number of double bonds in triglycerides were computed from acquired CSEI data [41], and the maps of MUFAs, PUFAs, and SFAs were subsequently calculated as a percentage of the total amount of lipids [42]. Tumour boundary was delineated in all slices of tumour regions of interest on the first echo of lipid composition images, with reference to diffusion-weighted and contrast-enhanced images. The peri-tumoural region was defined as a three-dimensional outward extension of 16 mm (8 voxels) from the tumour boundary (Supplementary Figure S3), consistent with our previous and other clinical breast imaging studies [25,26,43,44]. The whole breast was delineated to encompass only the adipose and fibroglandular tissue in *BRCA1/2* genetic mutation carriers and controls, with further exclusion of the tumour in women with breast cancer. The chest cavity and the subcutaneous fat were removed from images for all participants. The peri-tumoural and the whole breast lipid composition maps from a typical woman with breast cancer, and the whole breast lipid composition maps from a *BRCA1/2* genetic mutation carrier and a healthy control, are shown in Supplementary Figure S3. Adipose voxels with lipid signals over 60% of the total signal within the regions of interest were extracted from lipid composition maps, in accordance with our prior studies [25,26,45]. For highly skewed lipid composition distribution, median lipid constituents from the regions of interest were subsequently computed to reflect the central tendency.

### 4.3. Statistical Analysis

All statistical analyses were performed in R (v4.3.2, The R Foundation for Statistical Computing, Vienna, Austria). Wilcoxon rank sum tests were performed to compare the difference in lipid constituents in the whole breast of *BRCA1/2* genetic mutation carriers against women with breast cancer and controls. Wilcoxon signed rank paired tests were performed to compare the difference in lipid constituents between the peri-tumoural region and the whole breast in women with breast cancer. A two-sided *p* < 0.012 was considered statistically significant, after Bonferroni correction for 4-group comparisons. The coefficient of variation for intra-session repeatability was calculated as [standard deviation/mean] × 100%. The intraclass correlation coefficient for 6- and 12-month test–retest reliability was calculated using the ‘*irr*’ package based on the single-measurement, absolute-agreement, 2-way mixed-effects model [35]. An ICC value < 0.50 indicated poor reliability, 0.50–0.75 moderate reliability, 0.75–0.90 good reliability, and >0.90 excellent reliability.

## 5. Conclusions

In conclusion, lipid composition in *BRCA1/2* carriers showed deregulation compared to healthy controls but no significant difference to women with breast cancer. CSEI has good reproducibility, serving as a potential risk stratification and early detection tool for breast cancer.

## Figures and Tables

**Figure 1:**
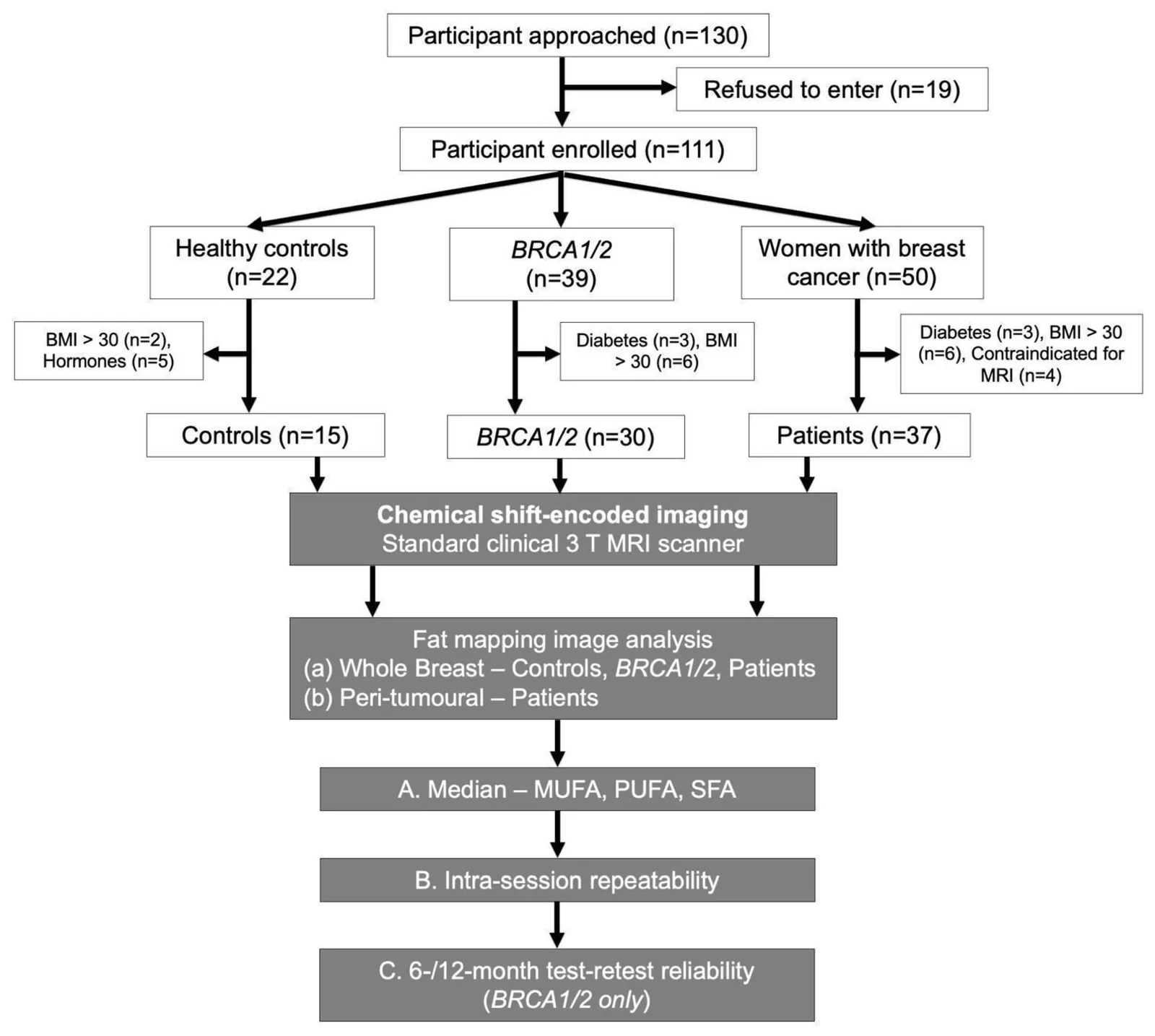
Study design. Eighty-two premenopausal female participants, 30 *BRCA1/2* genetic mutation carriers, 37 patients with invasive ductal carcinoma, and 15 healthy controls, took part in the study after meeting all the inclusion criteria. All participants underwent chemical shift-encoded imaging on a clinical 3 T MRI scanner.

**Figure 2:**
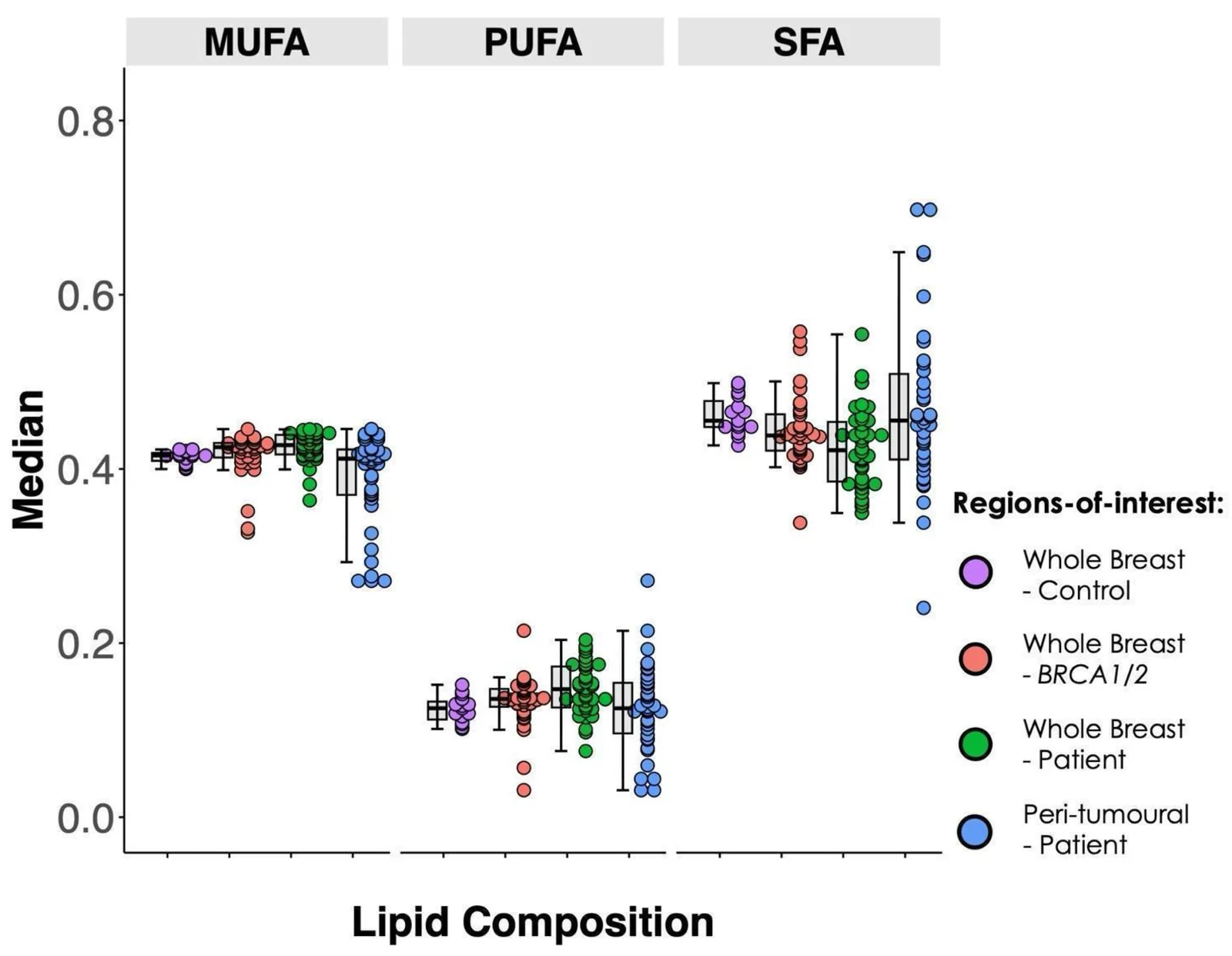
The group difference in median MUFAs, PUFAs, and SFAs in the whole breast of controls (WB-C) (n = 15), *BRCA1/2* genetic mutation carriers (WB-B) (n = 30), and patients with breast cancer (WB-P) (n = 37), and the peri-tumoural region in patients (Peri-P) (n = 37).

**Table 1:** Characteristics of study participants.

Characteristic	All (n = 82)	Controls (n = 15)	*BRCA1/2* (n = 30)	Patients (n = 37)	*p* Value
Age (years)	44.5 ± 7.9	42.7 ± 7.2	45.1 ± 8.3	45.6 ± 7.0	NS
Body mass index (BMI)	24.7 ± 3.5	23.8 ± 3.7	24.8 ± 3.3	26.0 ± 4.0	NS
IPAQ MET minutes	2555 ± 1105	2789 ± 1052	2498 ± 1145	2501 ± 1132	NS
*Breast density*					
Almost entirely fat	16	3	6	7	NS
Scattered fibroglandular	28	6	12	10	
Heterogeneous fibroglandular	35	4	12	19	
Extreme fibroglandular	3	2	0	1	

Note—descriptive statistics of *BRCA1/2* carriers, controls, and patients with breast cancer are shown for each group and the entire cohort. Unless otherwise noted, values are expressed as mean ± SD. One-way analysis of variance (ANOVA) and Fisher’s exact tests were conducted for group comparisons. IPAQ—International Physical Activity Questionnaire, MET—metabolic equivalent task, NS—not significant.

**Table 2:** Tumour histology in patients with breast cancer.

Tumour Histology	Patients (n = 37)
Tumour size (mm)	21.4 ± 5.8
*Histology grade*	
I	1 (2.7%)
II	21 (56.8%)
III	15 (40.5%)
*Lymphovascular invasion*	
Negative	32 (86.5%)
Positive	5 (13.5%)
*Molecular subtypes*	
Oestrogen receptor (ER+)	26 (70.3%)
Human epidermal growth factor receptor 2 (HER2+)	10 (27.0%)
Triple negative (TN)	11 (29.7%)

Note—histopathological findings for patients with breast cancer are shown, with quantitative data expressed as mean ± SD, and qualitative data expressed as number of positive observations and group percentage.

**Table 3:** Whole breast and peri-tumoural monounsaturated, polyunsaturated, and saturated fatty acids (MUFAs, PUFAs, SFAs) in healthy controls, *BRCA1/2* genetic mutation carriers, and patients with breast cancer.

Lipid	Controls (n = 15)	*BRCA1/2* (n = 30)	Patients (n = 37)		*WB-B* vs. WB-P	*WB-B* vs. WB-C	*WB-B* vs. Peri-P	WB-P vs. Peri-P
	Whole Breast (WB-C)	Whole Breast (*WB-B*)	Whole Breast (WB-P)	Peri-Tumoural (Peri-P)	*p* Value	*p* Value	*p* Value	*p* Value
	Median (IQR)	Median (IQR)	Median (IQR)	Median (IQR)				
*MUFAs*	0.41 (0.41–0.42)	0.42 (0.41–0.43)	0.43 (0.42–0.44)	0.41 (0.37–0.42)	0.10	0.01 *	0.02	0.008 *
*PUFAs*	0.13 (0.11–0.13)	0.14 (0.13–0.15)	0.15 (0.13–0.17)	0.13 (0.10–0.15)	0.06	0.03	0.31	0.008 *
*SFAs*	0.46 (0.45–0.48)	0.44 (0.42–0.46)	0.42 (0.39–0.45)	0.46 (0.41–0.51)	0.05	0.01 *	0.36	0.006 *

Note—the median lipid constituents were compared between healthy controls, *BRCA1/2* carriers, and patients with breast cancer. Wilcoxon rank sum tests were conducted for comparisons ‘*WB-B* vs. WB-P’, ‘*WB-B* vs. WB-C’, and ‘*WB-B* vs. Peri-P’. Wilcoxon signed rank paired tests were conducted for comparison ‘WB-P vs. Peri-P’. * Denotes a statistically significant difference after Bonferroni correction for 4-group comparisons (two-sided *p* < 0.012).

## Data Availability

The original contributions presented in this study are included in the article/Supplementary Materials. Further inquiries can be directed to the corresponding authors.

## Supplementary Materials

The following supporting information can be downloaded at: https://www.mdpi.com/article/10.3390/ijms27198472/s1.

## Abbreviations

The following abbreviations are used in this manuscript:

BRCA	BReast CAncer gene
CSEI	Chemical shift-encoded imaging
MUFAs	Monounsaturated fatty acids
PUFAs	Polyunsaturated fatty acids
SFAs	Saturated fatty acids
