# Supplementary material for: Lipid Composition in the Breast of Premenopausal BRCA1/2 Genetic Mutation Carriers Using MRI
Source: Int J Mol Sci. 2026 Sep 23;27(19):8472. doi: 10.3390/ijms27198472 (PMC13665113; doi:10.3390/ijms27198472)
Supplement: Supplementary file 1 [file ijms-27-08472-s001.zip › ijms-4480671-supplementary.pdf]

Supplementary Data

Supplementary Table S1. Intra-session repeatability (within-subject coefficient of variation, CoV) of monounsaturated, polyunsaturated and saturated fatty acids (MUFA, PUFA, SFA) in a subgroup of participants

Supplementary Figure S1. The group difference in coefficient of variation (CoV) in median MUFA, PUFA, SFA in the whole breast of controls, *BRCA1/2* genetic mutation carriers and patients with breast cancer, and the peri-tumoural region in patients.

Supplementary Figure S2. The longitudinal change in median MUFA, PUFA, SFA in the whole breast of *BRCA1/2* genetic mutation carriers between Visit 1 (V1, Baseline), Visit 2 (V2, 6-month) and Visit 3 (V3, 12-month).

Supplementary Figure S3. Lipid composition maps of the whole breast from a typical control, *BRCA1/2* genetic mutation carrier, patient with breast cancer, and the peri-tumoural region from the same patient.

Supplementary Table S1. Intra-session repeatability (within-subject coefficient of variation, CoV) of monounsaturated, polyunsaturated and saturated fatty acids (MUFA, PUFA, SFA) in a subgroup of participants

**Lipid**	**Measure**	**Whole Breast-Control (WB-C) (n=15)**		**Whole Breast-*BRCA1/2* (*WB-B*) (n=22)**		**Whole Breast-Patient (WB-P) (n=21)**		**Peri-tumoural-Patient (Peri-P) (n=21)**	
		Median (%)	*IQR (%)*	*Median (%)*	*IQR (%)*	*Median (%)*	*IQR (%)*	*Median (%)*	*IQR (%)*
MUFA	CoV	0.03	(0.02 – 0.17)	0.04	(0.00 – 0.18)	0.02	(0.01 – 0.06)	0.28	(0.05 – 0.59)
PUFA	CoV	0.12	(0.08 – 0.72)	0.14	(0.02 – 0.98)	0.17	(0.11 – 0.32)	1.25	(0.88 – 2.43)
SFA	CoV	0.06	(0.04 – 0.30)	0.07	(0.01 – 0.69)	0.09	(0.05 – 0.14)	0.60	(0.34 – 1.10)

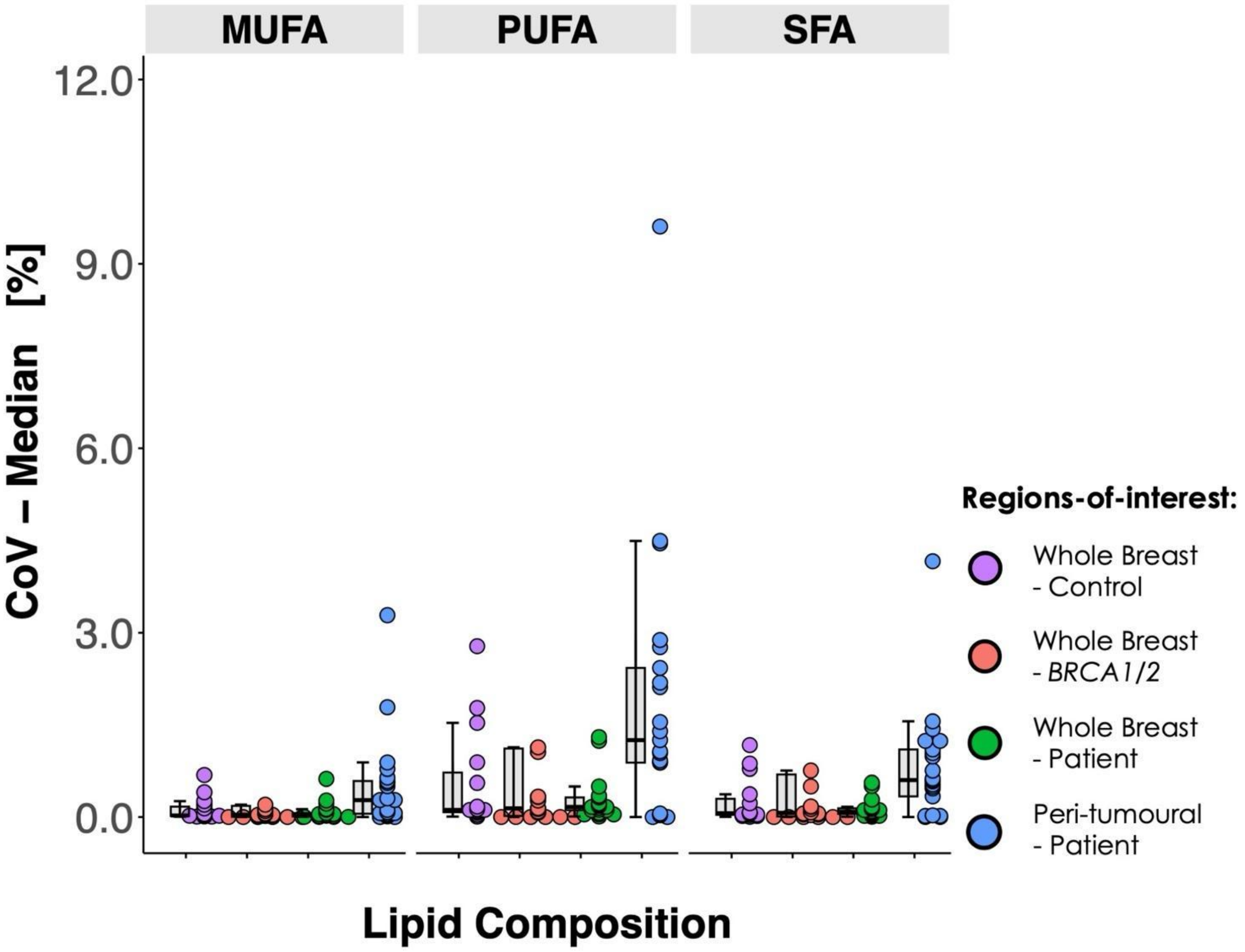

Supplementary Figure S1. The group difference in coefficient of variation (CoV) in median MUFA, PUFA, SFA in the whole breast of controls (WB-C) (n=15), *BRCA1/2* genetic mutation carriers (*WB-B*) (n=22) and patients with breast cancer (WB-P) (n=21), and the peri-tumoural region in patients (Peri-P) (n=21).

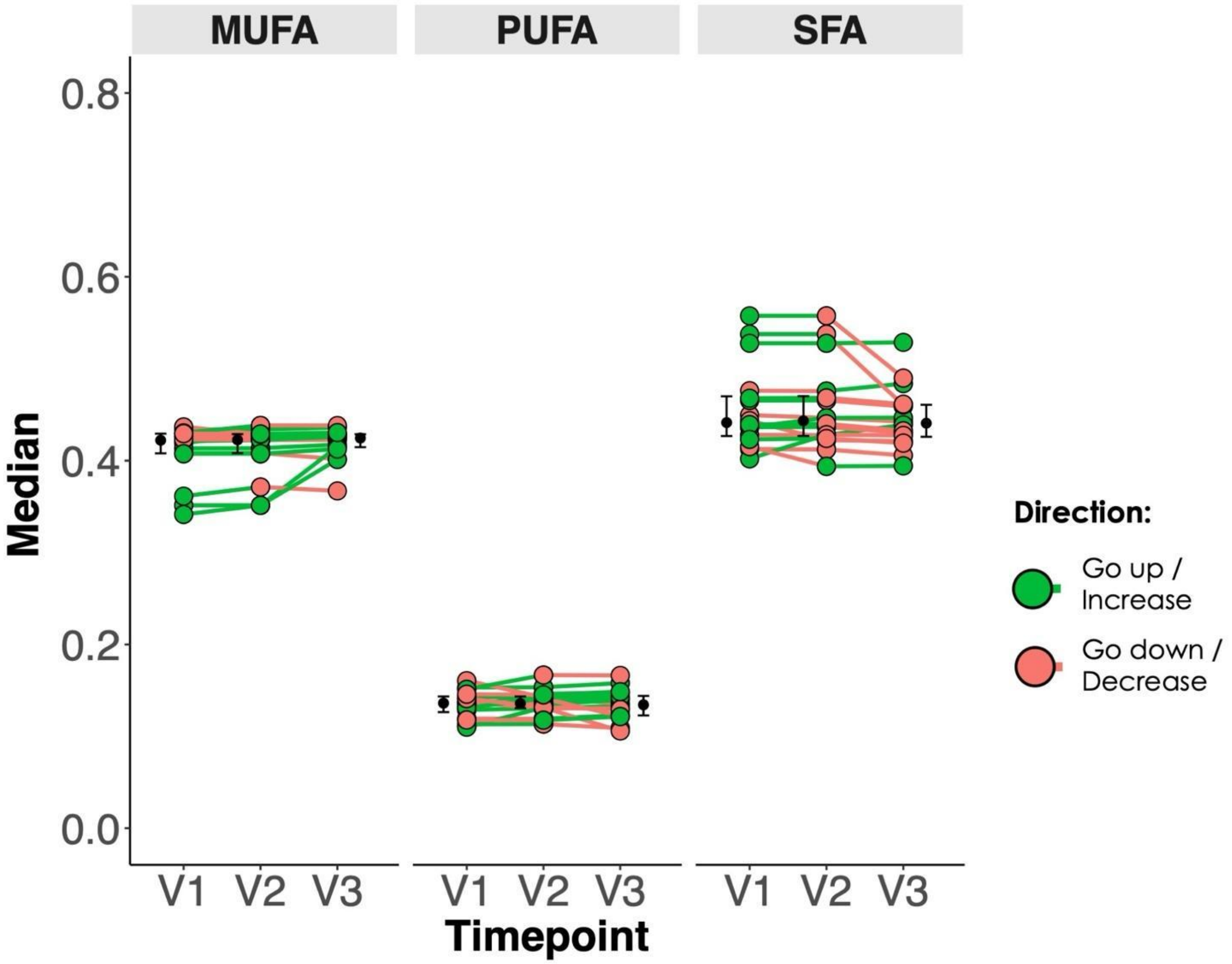

Supplementary Figure S2. The longitudinal change in median MUFA, PUFA, SFA in the whole breast of *BRCA1/2* genetic mutation carriers (n=15) between Visit 1 (V1, Baseline), Visit 2 (V2, 6-month) and Visit 3 (V3, 12-month).

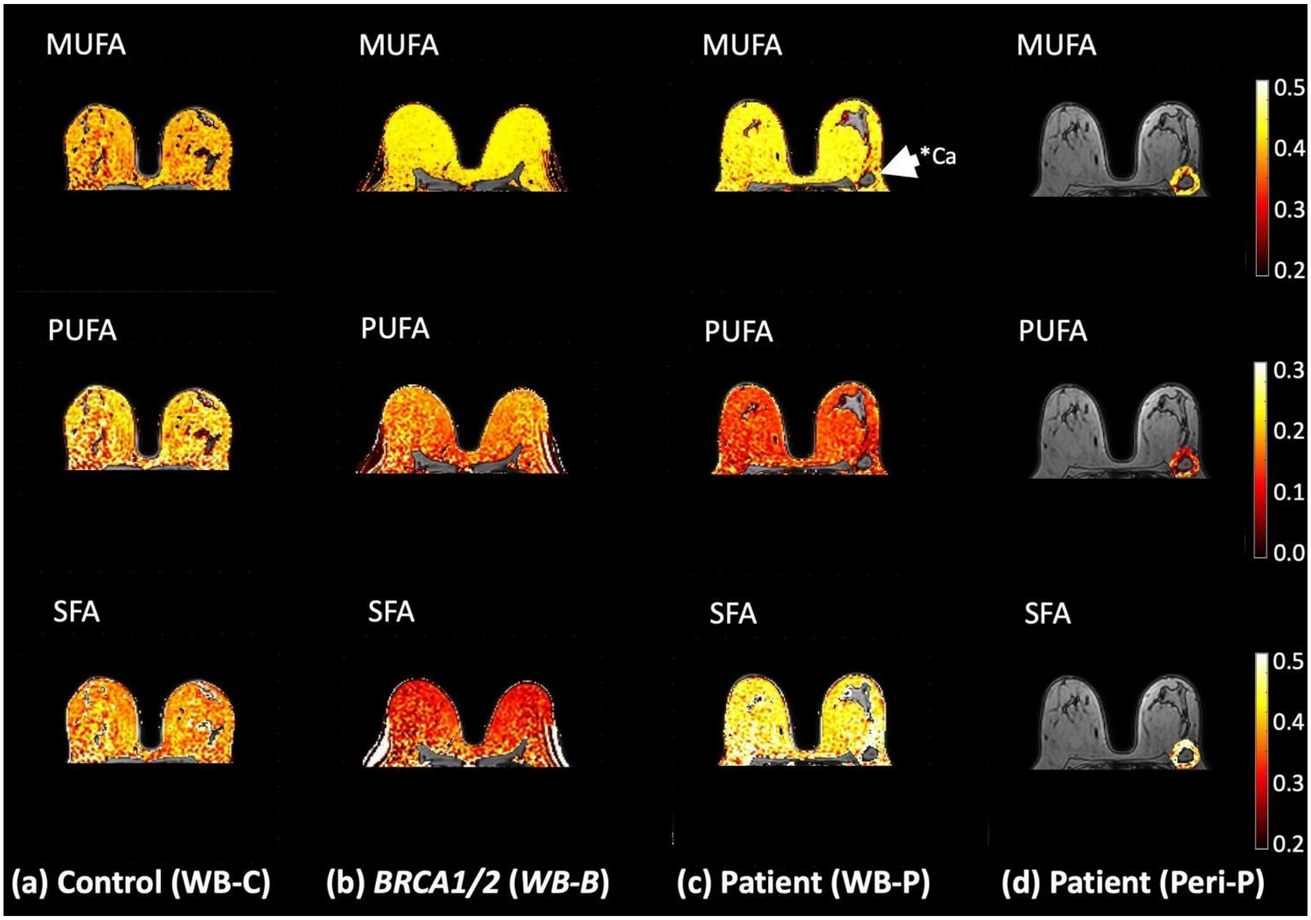

Supplementary Figure S3. Lipid composition maps of the whole breast from a typical (a) Control, (b) *BRCA1/2* genetic mutation carrier, (c) Patient with breast cancer, and (d) the peri-tumoural region from the same patient, overlaid on anatomical images. For the control and *BRCA1/2* carrier, the mid-thoracic slice is shown. For the patient, the slice at the greatest dimension of invasive ductal carcinoma (indicated by an arrow and *Ca) is shown.
